# Supplementary material for: Clinical and Product Features Associated with Outcome of DLBCL Patients to CD19-Targeted CAR T-Cell Therapy
Source: Cancers (Basel). 2021 Aug 25;13(17):4279. doi: 10.3390/cancers13174279 (PMC8428364; doi:10.3390/cancers13174279)
Supplement: Supplementary file 1 [file cancers-13-04279-s001.zip › cancers-1331994-SI.pdf]

cancers

MDPI

Article

Clinical and Product Features Associated with Outcome of DLBCL Patients to CD19-Targeted CAR T-Cell Therapy

Sylvain Lamure , François Van Laethem, Delphine De Verbizier, Claire Lozano, Eve Gehlkopf, Jean-Jacques Tudesq, Chris Serrand, Mehdi Benzaoui, Tarik Kanouni, Adeline Quintard, John De Vos, Emmanuelle Tchernonog, Laura Platon, Xavier Ayrignac, Patrice Ceballos, Anne Sirvent, Mickael François, Hanane Guedon, Philippe Quittet, Cedric Mongellaz, Aurélie Conte, Charles Herbaux, Caroline Bret, Naomi Taylor, Valérie Dardalhon and Guillaume Cartron

Table S1. Antibodies/detection reagents used for multiparametric flow cytometry analyses.

Antigen	Fluorochrome	Clone	Company
**Apheresis**			
Tetrachrome			Beckman Coulter
CD45	FITC	B3821F4A	
CD4	RD1	SFCI12T4D11	
CD8	ECD	SFCI21Thy2D3	
CD3	PC5	UCHT1	
CD16	PC7	3G8	Beckman Coulter
CD56	PC7	N901	Beckman Coulter
CD19	APC-A700	J3-119	Beckman Coulter
HLADR	PB	Immu-357	Beckman Coulter
LUCID DURACLONE Dry reagent			Beckman Coulter
CD31	FITC	5.6E	
CD197(CCR7)	PE	G043H7	
CD45RA	ECD	2H4	
CD25	PC5.5	B1,49,9	
CD45RO	PC7	UCHL1	
CD127	APC	R34.34	
CD8	APC-A700	B9.11	
CD3	APC-A750	UCHT1	
CD4	PB	13B8.2	
CD45	KRO	J33	
**CAR-T cell final product (bag)**			
CD19 CAR detection reagent		Cat# 130-115-965	Miltenyi Biotec
Streptavidin	PC7		Becton Dickinson
CD4	BD Horizon V450	Cat# 560345, RRID:AB_1645572	Becton Dickinson
CD8	APC Alexa eFluor780	Cat# 47-0088-41, RRID:AB_1272116	ThermoFisherScientific
CD45RA	BV711	Cat# 563733, RRID:AB_2738392	Becton Dickinson
CD45RO	BV650	Cat# 563749, RRID:AB_2744412	Becton Dickinson
CCR7	APC	Cat# 130-120-606, RRID:AB_2784048	Miltenyi Biotec
**Expansion of CAR+ T cells in the peripheral blood**			
CD19 CAR detection reagent		Cat# 130-115-965	Miltenyi Biotec
Anti-Biotin	PE	Cat# 130-110-951, REA746	Miltenyi Biotec
CD45	BV605	Cat# 564047, HI30	Becton Dickinson
CD3	BV510	Cat# 566779, OKT3	Becton Dickinson
CD4	APC R700	Cat# 564975, RPA-T4	Becton Dickinson
CD8	APC H7	Cat# 641400, SK1	Becton Dickinson
PD-1	BV421	Cat# 562516, EH12.1	Becton Dickinson
LAG3	FITC	Cat# 369308, 11C3C65	Biolegend
Tim3	PerCp Cy 5.5	Cat# 345016, F38-2^{E}2	Biolegend

Table S2. Univariate analysis of factors associated with PFS after CAR T-cells therapy.

	HR (IC95%)	*p*-value
Demography and lymphoma characteristics		
Age	0.99 (0.97-1.01)	0.4109
Gender (Female vs. Male)	0.32 (0.15-0.70)	0.0039
High-grade B-Lymphoma vs DLBCL	0.60 (0.21-1.71)	0.3409
t-FL vs DLBCL	0.89 (0.34-2.29)	0.7735
Bridging chemotherapy	1.77 (0.54-5.78)	0.3451
Number of treatment lines	1.26 (0.62-2.54)	0.5235
Characteristics at infusion		
TMTV	1.009 (1.001-1.016)	0.0222
Deauville scale	1.28 (0.78-2.09)	0.3254
Ann Arbor stage	1.51 (1.12-2.03)	0.0062
PS	1.47 (1.00-2.01)	0.0501
LDH	1.002 (1.001-1.004)	0.0002
aaIPI	2.11 (1.46-3.07)	<0.0001
CAR T-cells product (tisa-cel vs. Axi-cel)	2.34 (1.10-4.96)	0.0273
Apheresis composition		
Total number of mononucleated cells	0.94 (0.86-1.04)	0.2473
Total number of CD3	0.90 (0.74-1.10)	0.3220
%CD4 total	1.00 (0.98-1.02)	0.7875
%CD4 naive	1.00 (0.97-1.03)	0.8002
%CD4 naive 31^{+}	1.00 (0.97-1.03)	0.9643
%CD4 memory	1.00 (0.97-1.02)	0.8389
%CD4 T regulatory	0.95 (0.89-1.02)	0.1451
%CD8 total	1.00 (0.98-1.02)	0.9775
%CD8 naive	1.01 (0.97-1.02)	0.7666
%CD8 central memory	1.01 (0.97-1.01)	0.6268
%CD8 effector memory	0.99 (0.97-1.01)	0.1696
%CD8 EMRA	1.01 (0.99-1.03)	0.2184
%NK	0.99 (0.99-1.03)	0.4148

Abbreviations: CI Confidence Interval, DLBCL Diffuse Large B Cell Lymphoma, t-FL transformed Follicular Lymphoma, TMTV Total Metabolic Tumor Volume, PS *Performans Status*, LDH Lactate Dehydrogenase, aaIPI age adjusted international prognostic index, CAR T cell Chimeric Antigen Receptor T cell, EMRA Effector Memory expressing CD45RA.

Table S3. Baseline characteristics of patients according to the year of treatment.

	Year 1 (n=30)	Year 2 (n=30)	p-value
Demographic characteristics			
Age, years			
Median (range)	60 (47-66)	69 (39-73)	0.03
≥70, n (%)	3 (10)	15 (50)	<0.01
**Male gender**, n (%)	21 (70)	17 (57)	0.07
Lymphoma characteristics n (%)			
Histology			
DLBCL	21 (70)	22 (74)	<0.01
t-FL	6 (20)	4 (13)	0.25
High grade DLBCL	3 (10)	4 (13)	0.65
Number of previous line therapy			<0.01
≤2 lines	17 (57)	27 (90)	
>3 lines or more	13 (43)	3 (10)	
Autologous SCT	6 (20)	6 (20)	1
Bridging therapy, n (%)	27 (90)	27 (90)	1
Characteristics at infusion			
Ann Arbor Staging, n (%)			
No measurable disease	1 (3)	5 (17)	<0.01
I/II	5 (17)	13 (43)	<0.01
III/IV	24 (80)	12 (40)	0.18
LDH > normal limit, n (%)	14 (47)	11 (37)	0.20
PS 3-4, n (%)	4 (13)	1 (3)	0.02
Time between apheresis and infusion (days)	41 (34-70)	39 (30-174)	0.95
aaIPI, n (%)			
Low	3 (10)	13 (43)	<0.01
Intermediate-1	14 (47)	10 (33)	0.06
Intermediate-2	11 (37)	6 (20)	0.01
High	2 (7)	1 (3)	0.33

Abbreviations: DLBCL Diffuse Large B Cell Lymphoma, t-FL transformed Follicular Lymphoma, LDH Lactate Dehydrogenase, PS *Performans Status*, CAR T-cell Chimeric Antigen Receptor T-cell, SCT Stem Cell Transplantation, aaIPI age adjusted International Prognosis Index

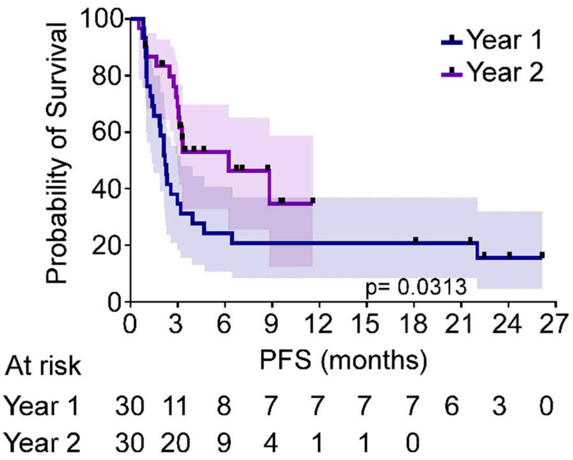

Figure S1. Progression free survival after CAR T-cell therapy as a function of the period of treatment. Progression Free Survival (PFS) of patients treated during the 1st and 2nd year of CAR T-cell activity at the CHU of Montpellier (n:30 per year). Response to treatment was analyzed via logistic regression; and time to death and time to progression were analyzed via univariate Cox proportional hazards models; p-value were calculated using a Wilcoxon Mann Whitney test.

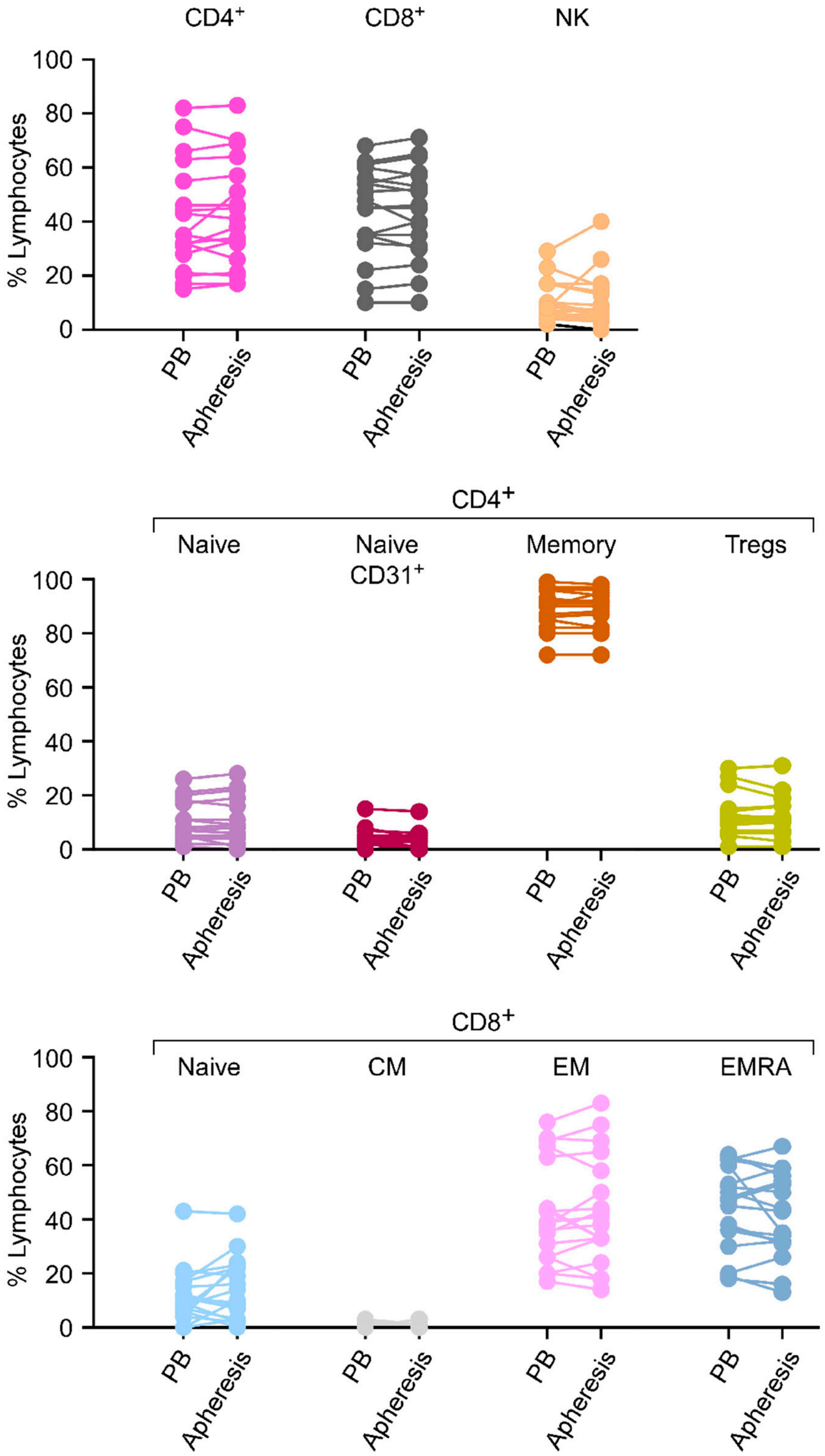

Figure S2. Comparison of T-cell subsets in peripheral blood and apheresis products of patients prior to CAR-T cell therapy. Comparison of the percentages of $CD4^+$, $CD8^+$ and NK cells (left) as well as T-cell subsets (Naive, Naive $CD31^+$, Memory, Treg) in peripheral blood (PB) and apheresis products of patients prior to CAR T-cell therapy (n:18), as evaluated by flow cytometry. p-values were calculated using Mann Whitney tests and no significant differences were detected.

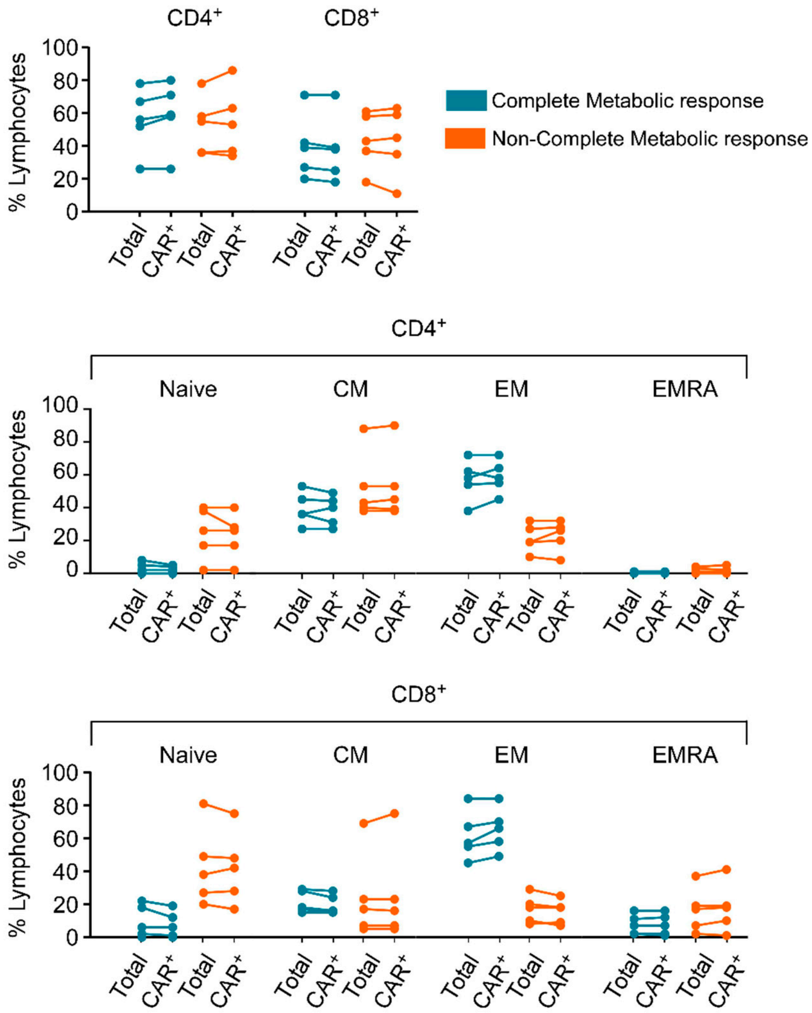

Figure S3. Comparison of the phenotype of all T-cells and the CAR+ subset in the commercial product as a function of clinical outcome. Percentages of CD4+ and CD8+ T-cells in the total commercial product as well as the CAR+ subset are presented for patients with a CMR (blue, n = 5) as compared to a non-complete response (orange, n = 5, left). CD4+ and CD8+ T-cell subsets phenotyped as naive, CM, EM, EMRA were evaluated in the total commercial product as well as the CAR+ subset as a function of clinical response. p-values were calculated using Mann Whitney tests and no significant differences were detected.

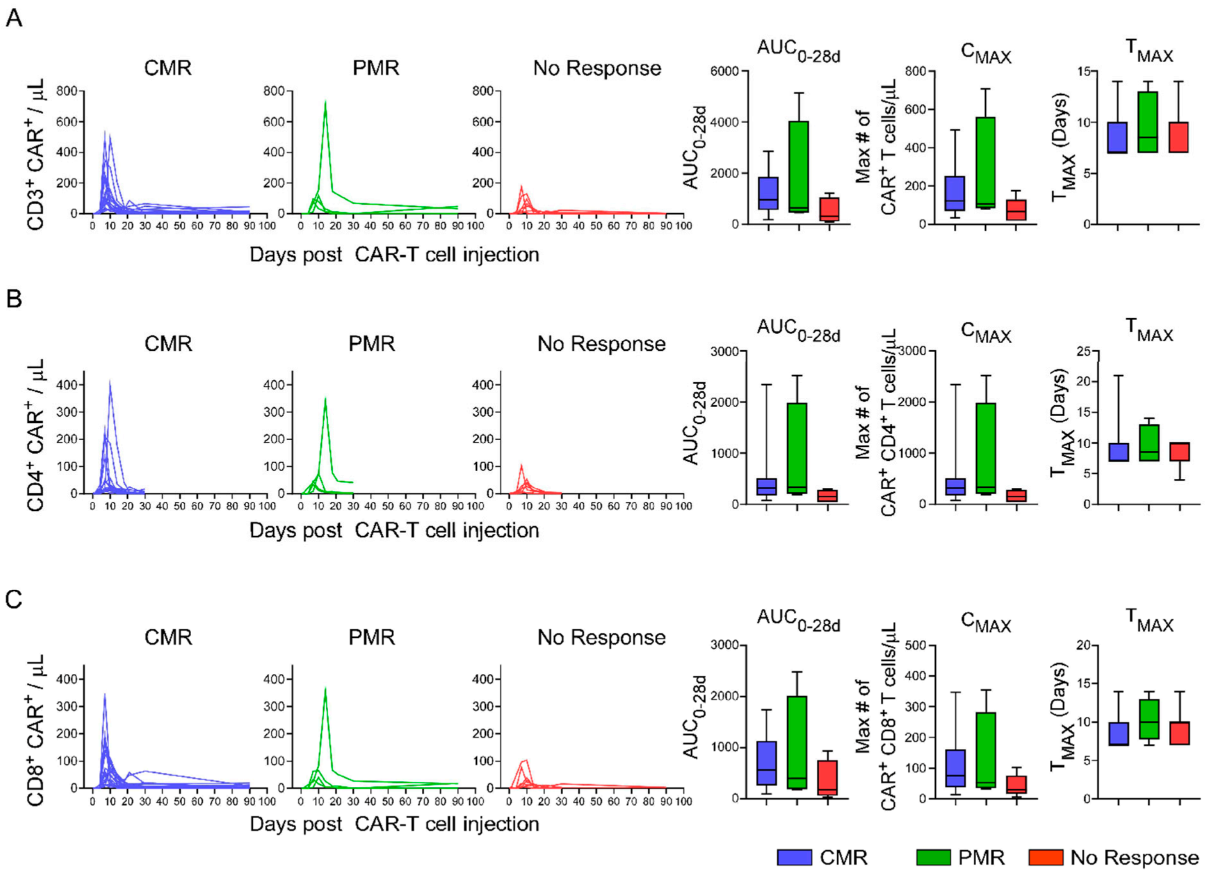

Figure S4. In vivo monitoring of the absolute number of CAR+ T-cells following CAR T-cell therapy. (**A**) The absolute number of CAR+ T-cells was monitored in the peripheral blood of 27 patients by flow cytometry at the indicated time points. Data are presented as a function of the clinical response; CMR, PMR, and no response (left graphs). Area under the curve (AUC_{0-28}), representing the absolute number of CAR+ T-cells between days 0 and day 28 following CAR+ T infusion, maximal concentration post-infusion (C_{MAX}) and time to maximal concentration (T_{MAX}) and their median, IQR and range (n:27) are presented. Similar analyses were performed for absolute numbers of CD4+CAR+ **(B)** and CD8+CAR+ (**C**) T cells. Note that patients with incomplete kinetics were not included in the AUC evaluation. p-values were calculated using Mann Whitney tests and no significant differences were detected.

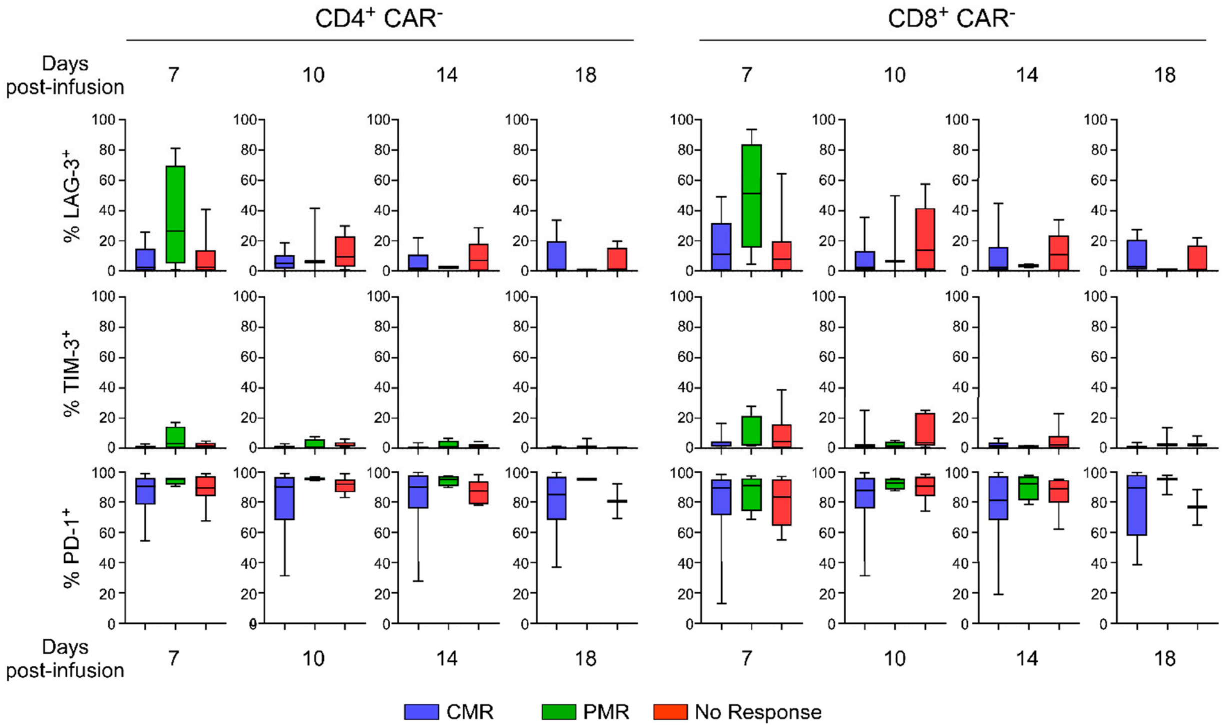

Figure S5. Evaluation of the exhaustion phenotype of CAR- T-cells as a function of clinical outcome. Exhaustion of CD4+CAR- (left) and CD8+CAR- (right) T-cells was evaluated from days 7 to 18 following CAR T infusion as a function of LAG3 (**A**) TIM-3 (**B**) and PD-1 (**C**) expression. Expression was assessed in patients with a CMR (n = 13), PMR (n = 4), and no response (n = 6) and their median, IQR and range are presented. p-values were calculated using Mann Whitney tests and no significant differences were detected.
